# Corneal Endothelial Changes Following Early Capsulotomy Using Neodymium:Yttrium–Aluminum–Garnet Laser

**DOI:** 10.3390/diagnostics12010150

**Published:** 2022-01-08

**Authors:** Hung-Chi Chen, Chia-Yi Lee, Chun-Fu Liu, Yi-Jen Hsueh, Yaa-Jyuhn James Meir, Chao-Min Cheng, Wei-Chi Wu

**Affiliations:** 1 Department of Ophthalmology, Chang Gung Memorial Hospital, Linkou 333423, Taiwan; legendlcf@gmail.com (C.-F.L.); t6612@seed.net.tw (Y.-J.H.); weichi666@gmail.com (W.-C.W.); 2 Department of Medicine, College of Medicine, Chang Gung University, Linkou 333423, Taiwan; 3 Center for Tissue Engineering, Chang Gung Memorial Hospital, Linkou 333423, Taiwan; 4 Department of Ophthalmology, Show Chwan Memorial Hospital, Changhua 50093, Taiwan; ao6u.3msn@hotmail.com; 5 Department of Ophthalmology, Chang Gung Memorial Hospital, Keelung 20401, Taiwan; 6 Program in Molecular Medicine, National Yang Ming Chiao Tung University, Hsinchu 30010, Taiwan; 7 Department of Biomedical Sciences, College of Medicine, Chang Gung University, Linkou 333423, Taiwan; hercats1@gmail.com; 8 Institute of Biomedical Engineering, National Tsing Hua University, Hsinchu 30010, Taiwan; chaomin@mx.nthu.edu.tw

**Keywords:** Nd:YAG laser capsulotomy, corneal endothelium, endothelial cell density, posterior capsular opacification

## Abstract

We aimed to survey whether the timing of neodymium:yttrium–aluminum–garnet (Nd:YAG) laser capsulotomy would alter the corneal endothelial morphology and density. A retrospective cohort study was conducted, and 48 patients with unilateral posterior capsular opacity (PCO) and Nd:YAG laser capsulotomy performance were enrolled. The participants were divided into the early Nd:YAG group (timing ≤ 12 months, *n* = 20) and late Nd:YAG group (timing > 12 months, *n*= 28) depending on elapsed months between phacoemulsification and Nd:YAG laser capsulotomy. Endothelial cell density (ECD), coefficient of variant (CV), hexagonality (HEX), and central corneal thickness (CCT) between the two groups were collected. A generalized estimate equation was conducted to evaluate the corneal endothelial parameters between the two groups with an adjusted odds ratio (aOR) and 95% confidence interval (CI). The CDVA was improved after treatment in both groups (both *p* < 0.001). Chronically, ECD in the early group was significantly decreased one week after treatment (2221.50 ± 327.73/mm^2^ vs. 2441.55 ± 321.80/mm^2^, *p* < 0.001), which recovered to 2369.95 ± 76.37/mm^2^ four weeks after the treatment but was still lower than the preoperative status (*p* < 0.001). In addition, the HEX percentage showed a significant reduction at four weeks after treatment (*p* = 0.028). The ECD in the early group was significantly lower than that in the late group (aOR: 0.167, 95% CI: 0.079–0.356, *p* = 0.003) in both week 1 (*p* < 0.001) and week 4 (*p* = 0.004) after laser treatment. In conclusion, the early application of Nd:YAG laser capsulotomy within one year after cataract surgery may be the reason for postoperative ECD decrement without known etiology.

## 1. Introduction

Posterior capsule opacification (PCO) is a late complication frequently encountered after cataract surgery [1,2], with an incidence of 1.2% and 13.2% in the first and fifth years postoperatively [3]. The incidence of PCO depends on the designs of intraocular lens (IOL) [4,5], despite the decreasing rates of PCO via the introduction of new IOL biomaterials as well as optic edge designs [6,7].

Currently, the development of PCO is routinely managed by the neodymium:yttrium–aluminum–garnet (Nd:YAG) laser capsulotomy with significant visual recovery in the majority of patients [8,9]. Although the Nd:YAG laser capsulotomy is effective, it is not free from prominent complications such as hyphema, transient intraocular pressure (IOP) elevation, cystoid macular edema, uveitis, dislocation of IOL, change of IOL position, and rhegmatogenous retinal detachment, particularly in the first few months [8,10,11,12].

Except for the above-mentioned complications, the Nd:YAG laser management could also affect the corneal endothelium with only limited regenerative capacity, and therefore, corneal decompensation with reduced vision is a major concern occasionally [13]. In previous studies, the laser iridotomy causes significant endothelial cell loss but without prominent corneal decompensation [14,15,16]. On the other hand, the Nd:YAG laser capsulotomy would contribute to the decrement of endothelial cell density (ECD), which is statistically insignificant [17,18,19]. Importantly, whether early or late arrangement of Nd:YAG laser capsulotomy exerts more harmful effects on the corneal endothelium was largely unknown. Moreover, the previous study that surveyed the influence of Nd:YAG laser capsulotomy on corneal endothelium only evaluated the ECD, while the changes of coefficient of variant (CV), hexagonality (HEX), and central corneal thickness (CCT) after Nd:YAG laser capsulotomy remain inconclusive.

The current study aimed to evaluate whether the timing of Nd:YAG laser capsulotomy would alter its influence on the corneal endothelium. Accordingly, the alterations of CV, HEX, and CCT after Nd:YAG laser capsulotomy with different elapsed times were investigated.

## 2. Materials and Methods

### 2.1. Subject Selection and Data Collection

A medical record review was performed in a tertiary hospital (Chang Gung Memorial Hospital), and patients were enrolled if they (1) received cataract surgery with a single-piece foldable hydrophobic acrylic IOL (SN60AT, Alcon, Texas, TX, USA) implantation because the rate of PCO is lesser in this IOL design [7,20], (2) received the management of Nd:YAG laser capsulotomy, and (3) were followed for at least four weeks after the Nd:YAG laser capsulotomy. On the other hand, several exclusion criteria were used to decrease vision-related confounding factors: (1) the diagnosis of prominent vitreoretinal disease throughout the study period including diabetic retinopathy, age-related macular degeneration, retinal vascular occlusion, macular hole, macular pucker, retinal detachment, vitreous hemorrhage, etc., (2) the diagnosis of glaucoma throughout the study period involving primary, secondary, open-angle, angle-closure, or normal tension glaucoma, (3) the receipt of corneal surgery throughout the study period such as photorefractive keratectomy, penetrating keratoplasty, laser in situ keratomileusis, (4) the development of posterior capsular rupture and vitreous loss during cataract surgery, and (5) the occurrence of IOL subluxation and dislocation during the follow-up period. After the selection process, these patients were divided into two groups according to the elapsed time (the time interval from cataract surgery to Nd:YAG laser capsulotomy) yielded the early-Nd:YAG group (elapsed timing less than 12 months, 20 patients) as well as the late-Nd:YAG group (elapsed timing more than 12 months, 28 patients).

### 2.2. Ophthalmic Examination

All participants received ophthalmic examination before Nd:YAG laser capsulotomy, one week, and four weeks after Nd:YAG laser capsulotomy and data of these ophthalmic examinations were obtained. The condition of IOL after Nd:YAG laser capsulotomy was also evaluated via slit-lamp biomicroscopy to ensure no subluxation or dislocation at each visit. For the visual function, the corrected-distance visual acuity (CDVA) was obtained from the Snellen chart and calculated as a logarithm of minimal angle of resolution (logMAR) in analysis. We converted Snellen chart visual acuity to logMAR via the following formula: LogMAR = LOG10(1/Snellen chart visual acuity). Pneumotonometry (Topcon c60, Topcon Corp, Tokyo, Japan) was used to measure the IOP for three times, and the averaged value was calculated. Regarding the corneal endothelial status, we used the non-contact in vivo specular microscope (CEM-530, Nidek, Gamagori, Japan) with nominal magnification of 400× to take photography of the corneal endothelium of each patient. The technician measured the corneal endothelium with focus on the central 3 mm of cornea before a photograph was obtained, and the obtained image was transmitted to the software program provided by the manufacturer, and therefore, the ECD, CV, HEX, and CCT were calculated. The technician would take additional endothelium photography if the primary picture was not clear enough to yield the four endothelial parameters. Regarding the accuracy of the specular microscope, our device was corrected every day in our hospital at the end of outpatient hours. The technician ensured the device yielded similar results 3 times when targeting the test film. In addition, this task is undertaken by two experienced technicians, both of whom have worked in this field for more than 10 years. The mean values of CDVA, IOP, ECD, CV, HEX, and CCT at each visit were enrolled in the subsequent analysis.

### 2.3. Statistical Analysis

All the statistical analyses in the current study were performed via the application of SPSS version 20.0 (SPSS Inc., Chicago, IL, USA). Descriptive analysis was used for the basic data including age, sex, the laterality of eye, total energy of Nd:YAG laser capsulotomy, elapsed time, and baseline ophthalmic parameters between the two groups. Then, the repeated one-way ANOVA was used to evaluate the difference of CDVA, IOP, ECD, CV, HEX, and CCT among the three visits in each group. To evaluate the influence of elapsed time on corneal endothelium, the generalized estimate equation was utilized to produce the adjusted odds ratio (aOR) with 95% confidence interval (CI) of the early-Nd:YAG group compared to the late Nd:YAG group concerning ECD, CV, HEX, and CCT. In addition, the major effects of age (older), sex (female), laterality (left), energy (higher total energy), CDVA (higher LogMAR, means worse visual acuity), and IOP (higher) were also incorporated into the GEE model to analyze the difference between the two groups on each endothelial parameter more precisely. A line chart was plotted to illustrate the trend of ECD change between the two groups. For the accuracy and repeatability of ECD measurement, the mean range of ECD deviation (maximum ECD–minimum ECD) for each participant in each measurement were calculated, and the Mann–Whitney U-test was adopted to compared the difference of ECD deviation between the two groups. A *p*-value lesser than 0.05 was regarded as statistically significant in the current study, and the *p*-value was depicted as *p* < 0.001 if a *p*-value less than 0.001 was found.

## 3. Results

The baseline characteristics of the study population are shown in Table 1. The mean age was 65.85 ± 8.47 in the Early Nd:YAG group and 67.21 ± 8.36 in the late Nd:YAG group without significant difference (*p* = 0.582), and the distribution of gender and laterality were also similar (both *p* > 0.05) (Table 1). The energy used in Nd:YAG laser capsulotomy was significantly higher in the early Nd:YAG group (69.36 ± 17.45 vs. 29.24 ± 18.30, *p* < 0.001), and the elapsed time was longer in the late Nd:YAG group (9.65 ± 1.42 vs. 16.03 ± 1.90, *p* < 0.001). The ophthalmic indexes, including CDVA, IOP, ECD, CV, HEX, and CCT, were similar at the initial presentation between the two groups (all *p* > 0.05) (Table 1).

The baseline CDVA was 0.75 ± 0.23 in the early Nd:YAG group, which significantly improved to 0.28 ± 0.07 one week after treatment and was maintained at 0.27 ± 0.08 four weeks after treatment (*p* < 0.001), while the IOP remained similar throughout the study period (*p* = 0.924). The ECD in the early Nd:YAG group was significantly decreased one week after the Nd:YAG laser capsulotomy compared to baseline status (2221.50 ± 327.73 vs. 2441.55 ± 321.80, *p* < 0.001), which recovered to 2369.95 ± 76.37 four weeks after the treatment but was still lower than the preoperative status (*p* < 0.001). On the other hand, the percentage of HEX showed a significant reduction at four weeks after the laser treatment (*p* = 0.028). The CV and CCT in the early Nd:YAG group did not change significantly (both *p* > 0.05) (Table 2). For the late Nd:YAG group, CDVA also improved from 0.75 ± 0.21 at baseline to 0.28 ± 0.08 at one week post-treatment and stayed at 0.29 ± 0.08 four weeks post-treatment (*p* < 0.001). The other parameters in the late Nd:YAG group including IOP, ECD, CV, HEX, and CCT were not influenced by the Nd:YAG laser capsulotomy (all *p* > 0.05) (Table 2). In addition, the deviation of ECD measurement was about 1–1.5% for each measurement, and the amount of deviation did not differ significantly between the two groups (*p* > 0.05).

**Figure 1:**
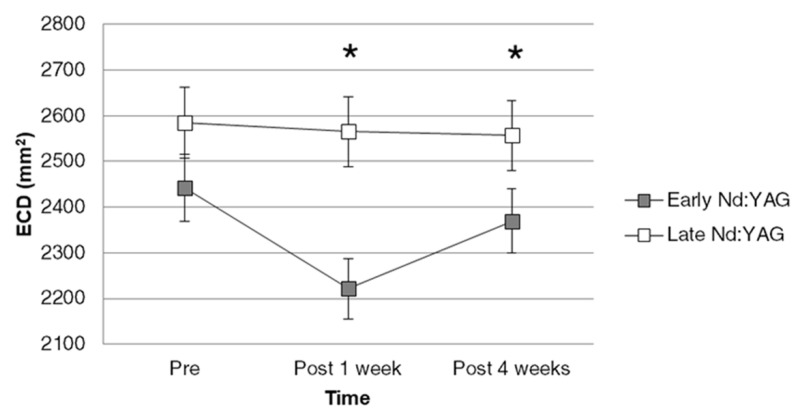
The ECD at different time points after early (<12 months) or late (≥12 months) Nd:YAG laser capsulotomy. Nd:YAG: neodymium:yttrium–aluminum–garnet; ECD: endothelial cell density; * denotes significant difference between the two groups.

Concerning the effect of elapsed time on the different corneal endothelial parameters, ECD in the early Nd:YAG group was significantly lower compared to the late Nd:YAG group after adjusting for demographic data, CDVA, and IOP (aOR: 0.167, 95% CI: 0.079–0.356, *p* = 0.003). Nevertheless, the change of CV, HEX, and CCT did not reveal any significant difference between the early Nd:YAG and late Nd:YAG groups (all *p* > 0.05) (Table 3). The trends of ECD shift after Nd:YAG laser capsulotomy between the two groups are demonstrated in Figure 1, and the difference of ECD between the two groups was mainly found at one week (*p* < 0.001) and four weeks (*p* = 0.004) after laser treatment according to the multivariable analysis. About the other parameters that affect the corneal endothelium, older age was associated with a lower CV value (aOR: 0.798, 95% CI: 0.666–0.957, *p* = 0.015). In addition, the worse CDVA was correlated with a lower HEX percentage (aOR: 0.042, 95% CI: 0.007–0.052, *p* = 0.018) and thicker CCT value (aOR: 1.555, 95% CI: 1.476–3.888, *p* = 0.002) (Table 3). On the other hand, the higher laser energy did not affect the ECD number significantly according to the results of the multivariable analysis (aOR: 1.961, 95%CI: 0.178–2.162, *p* = 0.215). Fortunately, there was no episode of corneal decompensation in both groups during the follow-up period.

## 4. Discussion

Briefly, the current study demonstrated that the CDVA had significant improvement after the Nd:YAG laser capsulotomy. However, a decreased ECD and HEX were observed in the early Nd:YAG group. In addition, the ECD was significantly lower in the early Nd:YAG group than the late Nd:YAG group in the multivariable analysis. The other factors that would influence the post-laser treatment corneal endothelium include age and CDVA.

Evidence has demonstrated a decrement of ECD after Nd:YAG laser capsulotomy, while the number of ECD decrement was regarded as insignificant [17,18,19]. In a previous study, about 2.3% of endothelial cell loss was observed, and no corneal decompensation was observed during the follow-up period [18]. Another retrospective study also showed a similar result that the ECD dropped with a mild number of 1.5% [19]. Nevertheless, the previous studies did not consider the effect of elapsed time of Nd:YAG laser capsulotomy, and only one study recorded the mean elapsed time, which was 24 months [18]. Transient corneal edema and bullous keratopathy often developed in the early postoperative period of cataract surgery [21,22], and the laser iridotomy also using laser energy would lead to bullous keratopathy in the general population upon damage to the corneal endothelium [23]. Consequently, although the energy used in Nd:YAG laser capsulotomy is much lower than that in laser iridotomy [24,25], the application of Ndya laser onto the early-postoperative cornea may add additional stress to the vulnerable cornea and contribute to endothelial injury. In the current study, the early Nd:YAG group who received Nd:YAG laser capsulotomy within one year of cataract surgery showed a significantly lower ECD compared to those who received the same treatment at least one year after the cataract surgery, which may be preliminary experience to our knowledge. Compared to the previous research [17,18,19], the advantages of the current study include the multiple measurements of ECD and the use of multivariable analysis to erase possible confounders. Although there was no severe corneal injury such as the bullous keratopathy or corneal opacity in the early Nd:YAG group throughout the study interval, the maximum loss of nearly 10% of ECD may still lead to certain endothelial damage compared to the late-treatment counterparts, especially for those with impaired endothelium status preoperatively. In addition, the numbers of ECD were insignificantly lower in the early group than that in the late group, given that the ECD would decrease in the early postoperative period and recover slightly one year after cataract surgery [26]. Consequently, the application of Nd:YAG laser to patients in the early postoperative period with relatively lower ECD count may damage the corneal endothelium more prominently. Except for the early-applied Nd:YAG laser capsulotomy, the worse CDVA also correlated to lower ECD, in which the poor CDVA may imply a worse corneal condition; thus, the ECD would decrease more easily.

Regarding the change of other corneal endothelial parameters after Nd:YAG laser capsulotomy, such an issue was rarely addressed previously. In the current study, the change of CV, HEX, and CCT after Nd:YAG laser capsulotomy did not show any significant difference between the two groups. Accordingly, the elapsed time of Nd:YAG laser capsulotomy did not seem to influence the corneal morphology and corneal thickness to a large extent. However, the HEX decreased progressively, and the CCT increased gradually in the early Nd:YAG group compared to the steady value of the two parameters in the late Nd:YAG group. Perhaps a significant difference of HEX and CCT would develop between the two groups with a longer follow-up interval, but the results of the current study cannot fully support this speculation. In addition to the elapsed time of Nd:YAG laser capsulotomy, the older age was correlated to a higher value of CV. In a previous study, the higher CV was also associated with older age, which implies a suboptimal polymegathism [27], and the findings of the current study corresponded with previous experience. In addition, the worse CDVA was related to a lower HEX percentage and higher CCT value, which may be because the impaired corneal condition will also lead to a decrease of vision.

For the alteration of ophthalmic parameters between baseline and after Nd:YAG laser capsulotomy, the CDVA were improved in the both groups, in which the reduced visual acuity caused via PCO is the major indication to perform Nd:YAG laser capsulotomy [8,28]. The IOP, on the contrary, did not reveal any significant change in both groups. Transient elevation of IOP has been observed in patients receiving Nd:YAG laser capsulotomy [29]. Still, the elevated IOP after Nd:YAG laser capsulotomy often returns back to the normal range within days with the application of anti-glaucomatous medications [30]. After Nd:YAG laser capsulotomy, brimonidine was routinely prescribed for our patients; thus, the post-treatment IOP control may be adequate according to previous experience [31]. In addition, the first time we measured IOP was one week after the Nd:YAG laser capsulotomy; thus, a persistent IOP was relatively less encountered at that time point [30]. Regarding the corneal parameters, the ECD in the early Nd:YAG group dropped significantly after Nd:YAG laser capsulotomy and did not recover to baseline value four weeks after the management. In addition, the HEX percentage four weeks after treatment was lower compared to the percentage in pre-treatment status and one week after treatment. On the other hand, all the four corneal parameters remain unchanged throughout the study period in the late Nd:YAG group. These findings may further state that although the shift of HEX percentage was insignificant in between-group analysis, the early application of Nd:YAG laser capsulotomy could result in general damage of the corneal endothelium. Interestingly, a significant recovery of ECD was observed four weeks post-treatment in the early Nd:YAG group compared to the ECD one week after the laser treatment. We attribute the “significant loss and subsequent recovery” in the early Nd:YAG group to the following speculations. First, from our unpublished data of ECD in patients after phacoemulsification, we found that the ECD tremendously declines in the first 6 months postoperative, particularly in the first month, which then resumes to a higher level in the long-term (>6 months) follow-up. This “rebounding phenomenon” could feasibly be applied to the scenario in the post-YAG laser. However, the rebounding phenomenon was not obvious in the late treatment group, which was probably due to less energy being consumed during the procedure. To test this hypothesis, we would initiate another study, including patients either receiving phacoemulsification or YAG laser procedures in the future.

The demographic data and baseline characteristics between the early Nd:YAG group and the late Nd:YAG group were grossly similar, including the age, gender, laterality, and the four corneal parameters. The elapsed times between the two groups were significantly different because of our grouping process. Interestingly, the energy delivered during the Nd:YAG laser capsulotomy was significantly higher in the early Nd:YAG group than that in the late Nd:YAG group. Although subtypes of PCO are determinant to the energy levels of capsulotomy [25,32], it remains unknown whether early-formed PCO are prone to be fibrous type or not. A possible explanation for the higher laser energy in the early Nd:YAG group is that the early Nd:YAG group in our study indeed presented a more dense/fibrous PCO, which was recorded in 4 patients in the early Nd:YAG group, while no dense PCO was documented in the late Nd:YAG group; thus, the physician may decide to perform Nd:YAG laser earlier, and the laser energy was much greater. Still, further research is needed to survey whether early visual-significant PCO tend to be more severe. However, based on the findings of the current study, the corneal endothelium may benefit from a later arrangement of Nd:YAG laser capsulotomy. Consequently, the physician may make a meticulous evaluation of the cornea for the patient before the performance of Nd:YAG laser capsulotomy in the early postoperative period.

There are still some limitations in the current study. Firstly, the retrospective nature of the current study could increase the heterogeneity between the study and control groups compared to a prospective one. In addition, the patient number in the whole study population was only 48 cases and would lead to statistical bias even in multivariable analysis. In addition, the follow-up period is inadequate with only four weeks post-treatment. Since we did not perform specular microscope examination periodically for patients after cataract surgery, the subsequent corneal endothelium condition cannot be assessed. Due to the retrospective design, a meticulous PCO grading system is absent in the current study, which may make the analysis less accurate. Finally, the higher total energy delivery of Nd:YAG laser capsulotomy in the early Nd:YAG group may also cause corneal endothelial damage and lower ECD in that group [26,33]. Still, the higher energy did not correlate to the change of any corneal parameters; thus, the defected corneal condition in the early Nd:YAG group might result from the early application of a laser rather than higher total laser energy.

## 5. Conclusions

In conclusion, the early application of Nd:YAG laser capsulotomy within one year after the cataract surgery may contribute to significantly decreased ECD compared to those receiving Nd:YAG laser capsulotomy at least one year postoperatively. Furthermore, the ECD and HEX values were prominently deteriorated compared to the baseline value in those that received early Nd:YAG laser capsulotomy. Consequently, the arrangement of early Nd:YAG laser capsulotomy could be included in the differential diagnosis of postoperative ECD decrement without other prominent etiology, and early Nd:YAG laser capsulotomy should be avoided in patients with guarded postoperative corneal status. Further large-scale prospective research to evaluate the long-term effect of Nd:YAG laser capsulotomy on the corneal endothelium is mandatory.

## Figures and Tables

**Table 1:** Baseline demographic and clinical characteristics of patients undergoing Nd:YAG laser capsulotomy.

Characteristics	Early Nd:YAG Group (<12 Months, *n* = 20)	Late Nd:YAG Group (≥12 Months, *n* = 28)	*p*-Value
Age (year, mean ± SD)	65.85 ± 8.47	67.21 ± 8.36	0.582
Gender (male/female)	8:12	9:19	0.760
Laterality (right/left)	12:8	11:17	0.241
Energy (mJ, mean ± SD)	69.36 ± 17.45	29.24 ± 18.30	<0.001 *
Elapsed time (months)	9.65 ± 1.42	16.03 ± 1.90	<0.001 *
CDVA	0.77 ± 0.23	0.75 ± 0.21	0.708
IOP (mmHg)	15.89 ± 2.85	15.56 ± 3.48	0.731
ECD (mm^2^)	2441.55 ± 321.80	2584.57 ± 274.51	0.070
CV (%)	30.00 ± 7.91	31.57 ± 11.58	0.602
HEX (%)	54.40 ± 14.54	54.75 ± 11.11	0.925
CCT (µm)	543.85 ± 32.89	531.29 ± 35.95	0.223

Nd:YAG: neodymium:yttrium–aluminum–garnet; N: number; SD: standard deviation; CDVA: corrected distance visual acuity; IOP: intraocular pressure; ECD: endothelial cell count; CV: coefficient of variant; HEX: hexagonality; CCT: central corneal thickness; * denotes significant difference between the two groups.

**Table 2:** Change of ocular and corneal parameters after early or late Nd:YAG laser capsulotomy.

Parameters	Pre-Nd:YAG	Post-Nd:YAG 1 Week	Post-Nd:YAG 4 Weeks	*p*-Value
Early Nd:YAG group (≤12 months, *n* = 20)				
CDVA	0.75 ± 0.23 ^A^	0.28 ± 0.07 ^B^	0.27 ± 0.08 ^B^	<0.001 *
IOP (mmHg)	15.89 ± 2.85	16.24 ± 3.25	16.05 ± 3.36	0.924
ECD (mm^2^)	2441.55 ± 321.80 ^A^	2221.50 ± 327.73 ^C^	2369.95 ± 341.53 ^B^	<0.001 *
CV (%)	30.00 ± 7.91	32.90 ± 12.26	28.65 ± 7.29	0.249
HEX (%)	54.40 ± 14.54 ^A^	50.45 ± 15.99 ^A^	48.10 ± 14.98 ^B^	0.028 *
CCT (µm)	543.85 ± 32.89	550.55 ± 35.37	561.60 ± 42.06	0.239
Late Nd:YAG group (>12 months, *n* = 28)				
CDVA	0.75 ± 0.21 ^A^	0.28 ± 0.08 ^B^	0.29 ± 0.08 ^B^	<0.001 *
IOP (mmHg)	15.56 ± 3.48	15.22 ± 3.50	14.60 ± 3.26	0.564
ECD (mm^2^)	2584.57 ± 274.51	2565.14 ± 304.66	2557.32 ± 317.87	0.100
CV (%)	31.57 ± 11.58	30.29 ± 6.34	27.57 ± 6.72	0.128
HEX (%)	54.75 ± 11.11	51.82 ± 17.43	55.00 ± 12.90	0.436
CCT (µm)	531.29 ± 35.95	546.57 ± 47.27	546.11 ± 30.13	0.184

Nd:YAG: neodymium:yttrium–aluminum–garnet; CDVA: corrected distance visual acuity; IOP: intraocular pressure; ECD: endothelial cell density; CV: coefficient of variant; HEX: hexagonality; CCT: central corneal thickness; * denotes significant difference among the three periods; ^A^^,^^B^^,^^C^: intergroup comparison, the same letter represents no significant difference among groups.

**Table 3:** The effect of timing of Nd:YAG on corneal endothelial parameters.

Parameters	aOR	95% CI		*p*-Value
		Lower	Upper	
ECD				
Early Nd:YAG	0.167	0.079	0.356	0.003 *
Age (year)	7.536	0.001	47.709	0.444
Sex	5.754	0.145	9.209	0.765
Laterality	1.576	0.712	3.349	0.695
Energy (mJ)	1.961	0.178	2.162	0.215
CDVA	2.201	1.205	4.021	<0.001 *
IOP (mmHg)	0.406	0.003	1.615	0.725
CV				
Early Nd:YAG	1.826	0.323	10.308	0.158
Age (year)	0.798	0.666	0.957	0.015 *
Sex	1.654	0.493	5.549	0.117
Laterality	0.898	0.047	1.728	0.943
Energy (mJ)	0.943	0.888	1.002	0.058
CDVA	3.649	0.020	6.650	0.626
IOP (mmHg)	0.937	0.664	1.321	0.709
HEX				
Early Nd:YAG	0.793	0.235	2.670	0.551
Age (year)	1.404	0.602	3.276	0.432
Sex	0.736	0.001	3.105	0.396
Laterality	0.008	0.001	3.118	0.530
Energy (mJ)	0.979	0.651	1.471	0.918
CDVA	0.042	0.007	0.052	0.018 *
IOP (mmHg)	0.229	0.036	1.461	0.119
CCT				
Early Nd:YAG	3.431	0.001	6.647	0.787
Age (year)	0.952	0.621	1.461	0.822
Sex	1.669	0.001	2.351	0.890
Laterality	0.020	0.003	1.774	0.073
Energy (mJ)	0.864	0.741	1.007	0.061
CDVA	1.555	1.476	3.888	0.002 *
IOP (mmHg)	1.184	0.714	1.965	0.512

aOR: adjusted odds ratio; CI: confidence interval; Nd:YAG: neodymium:yttrium–aluminum–garnet; CDVA: corrected distance visual acuity; IOP: intraocular pressure; ECD: endothelial cell density; CV: coefficient of variant; HEX: hexagonality; CCT: central corneal thickness; * denotes significant correlation to corneal endothelial parameters.

## Data Availability

The data will be available upon reasonable request.

## References

[1] Wormstone I.M., Wang L., Liu C.S. (2009). Posterior capsule opacification. Exp. Eye Res..

[2] Dewey S. (2006). Posterior capsule opacification. Curr. Opin. Ophthalmol..

[3] Lindholm J.M., Laine I., Tuuminen R. (2019). Five-year cumulative incidence and risk factors of nd:Yag capsulotomy in 10 044 hydrophobic acrylic 1-piece and 3-piece intraocular lenses. Am. J. Ophthalmol..

[4] Bilbao-Calabuig R., Llovet-Osuna F., González-López F., Beltrán J. (2016). Nd:Yag capsulotomy rates with two trifocal intraocular lenses. J. Refract. Surg..

[5] Thom H., Ender F., Samavedam S., Perez Vivez C., Gupta S., Dhariwal M., de Haan J., O’Boyle D. (2019). Effect of acrysof versus other intraocular lens properties on the risk of nd:Yag capsulotomy after cataract surgery: A systematic literature review and network meta-analysis. PLoS ONE.

[6] Cheng J.W., Wei R.L., Cai J.P., Xi G.L., Zhu H., Li Y., Ma X.Y. (2007). Efficacy of different intraocular lens materials and optic edge designs in preventing posterior capsular opacification: A meta-analysis. Am. J. Ophthalmol..

[7] Li Y., Wang J., Chen Z., Tang X. (2013). Effect of hydrophobic acrylic versus hydrophilic acrylic intraocular lens on posterior capsule opacification: Meta-analysis. PLoS ONE.

[8] Aslam T.M., Devlin H., Dhillon B. (2003). Use of nd:Yag laser capsulotomy. Surv. Ophthalmol..

[9] Yotsukura E., Torii H., Saiki M., Negishi K., Tsubota K. (2016). Effect of neodymium: Yag laser capsulotomy on visual function in patients with posterior capsule opacification and good visual acuity. J. Cataract Refract. Surg..

[10] Wesolosky J.D., Tennant M., Rudnisky C.J. (2017). Rate of retinal tear and detachment after neodymium: Yag capsulotomy. J. Cataract Refract. Surg..

[11] Achiron A. (2017). Intraocular pressure spikes following neodymium-doped yttrium aluminum garnet laser capsulotomy: Current prevalence and management in israel. J. Curr. Glaucoma Pract..

[12] Findl O., Drexler W., Menapace R., Georgopoulos M., Rainer G., Hitzenberger C.K., Fercher A.F. (1999). Changes in intraocular lens position after neodymium: Yag capsulotomy. J. Cataract Refract. Surg..

[13] Hoppenreijs V.P., Pels E., Vrensen G.F., Treffers W.F. (1996). Corneal endothelium and growth factors. Surv. Ophthalmol..

[14] Wang P.X., Koh V.T., Loon S.C. (2014). Laser iridotomy and the corneal endothelium: A systemic review. Acta Ophthalmol..

[15] Wu S.C., Jeng S., Huang S.C., Lin S.M. (2000). Corneal endothelial damage after neodymium: Yag laser iridotomy. Ophthalmic Surg. Lasers.

[16] Kozobolis V.P., Detorakis E.T., Vlachonikolis I.G., Pallikaris I.G. (1998). Endothelial corneal damage after neodymium: Yag laser treatment: Pupillary membranectomies, iridotomies, capsulotomies. Ophthalmic Surg. Lasers.

[17] Wasserman E.L., Axt J.C., Sheets J.H. (1985). Neodymium: Yag laser posterior capsulotomy. J. Am. Intraocul. Implant Soc..

[18] Slomovic A.R., Parrish R.K., Forster R.K., Cubillas A. (1986). Neodymium-yag laser posterior capsulotomy. Central corneal endothelial cell density. Arch. Ophthalmol..

[19] Kraff M.C., Sanders D.R., Lieberman H.L. (1985). Intraocular pressure and the corneal endothelium after neodymium-yag laser posterior capsulotomy. Relative effects of aphakia and pseudophakia. Arch. Ophthalmol..

[20] Auffarth G.U., Brezin A., Caporossi A., Lafuma A., Mendicute J., Berdeaux G., Smith A.F. (2004). Comparison of nd:Yag capsulotomy rates following phacoemulsification with implantation of pmma, silicone, or acrylic intra-ocular lenses in four european countries. Ophthalmic Epidemiol..

[21] Sharma N., Singhal D., Nair S.P., Sahay P., Sreeshankar S.S., Maharana P.K. (2017). Corneal edema after phacoemulsification. Indian J. Ophthalmol..

[22] Behndig A., Lundberg B. (2002). Transient corneal edema after phacoemulsification: Comparison of 3 viscoelastic regimens. J. Cataract Refract. Surg..

[23] Ang L.P., Higashihara H., Sotozono C., Shanmuganathan V.A., Dua H., Tan D.T., Kinoshita S. (2007). Argon laser iridotomy-induced bullous keratopathy a growing problem in japan. Br. J. Ophthalmol..

[24] Lim L., Seah S.K., Lim A.S. (1996). Comparison of argon laser iridotomy and sequential argon laser and nd:Yag laser iridotomy in dark irides. Ophthalmic Surg. Lasers.

[25] Bhargava R., Kumar P., Phogat H., Chaudhary K.P. (2015). Neodymium-yttrium aluminium garnet laser capsulotomy energy levels for posterior capsule opacification. J. Ophthalmic Vis. Res..

[26] Storr-Paulsen A., Norregaard J.C., Ahmed S., Storr-Paulsen T., Pedersen T.H. (2008). Endothelial cell damage after cataract surgery: Divide-and-conquer versus phaco-chop technique. J. Cataract Refract. Surg..

[27] Tananuvat N., Khumchoo N. (2020). Corneal thickness and endothelial morphology in normal thai eyes. BMC Ophthalmol..

[28] Awasthi N., Guo S., Wagner B.J. (2009). Posterior capsular opacification: A problem reduced but not yet eradicated. Arch. Ophthalmol..

[29] Shetty N.K., Sridhar S. (2016). Study of variation in intraocular pressure spike (iop) following nd-yag laser capsulotomy. J. Clin. Diagn. Res..

[30] Artunay O., Yuzbasioglu E., Unal M., Rasier R., Sengul A., Bahcecioglu H. (2010). Bimatoprost 0.03% versus brimonidine 0.2% in the prevention of intraocular pressure spike following neodymium: Yttrium–aluminum–garnet laser posterior capsulotomy. J. Ocul. Pharmacol. Ther..

[31] Seong G.J., Lee Y.G., Lee J.H., Lim S.J., Lee S.C., Hong Y.J., Kwon O.W., Kim H.B. (2000). Effect of 0.2% brimonidine in preventing intraocular pressure elevation after nd:Yag laser posterior capsulotomy. Ophthalmic Surg. Lasers.

[32] Bhargava R., Kumar P., Prakash A., Chaudhary K.P. (2012). Estimation of mean nd:Yag laser capsulotomy energy levels for membranous and fibrous posterior capsular opacification. Nepal. J. Ophthalmol..

[33] Zabel R.W., MacDonald I.M., Mintsioulis G. (1991). Corneal endothelial decompensation after argon laser iridotomy. Can. J. Ophthalmol..

